# Supplementary figures and images for: Aging and sperm signals alter DNA break formation and repair in the C. elegans germline
Source: PLoS Genet. 2022 Nov 7;18(11):e1010282. doi: 10.1371/journal.pgen.1010282 (PMC9671421; doi:10.1371/journal.pgen.1010282)

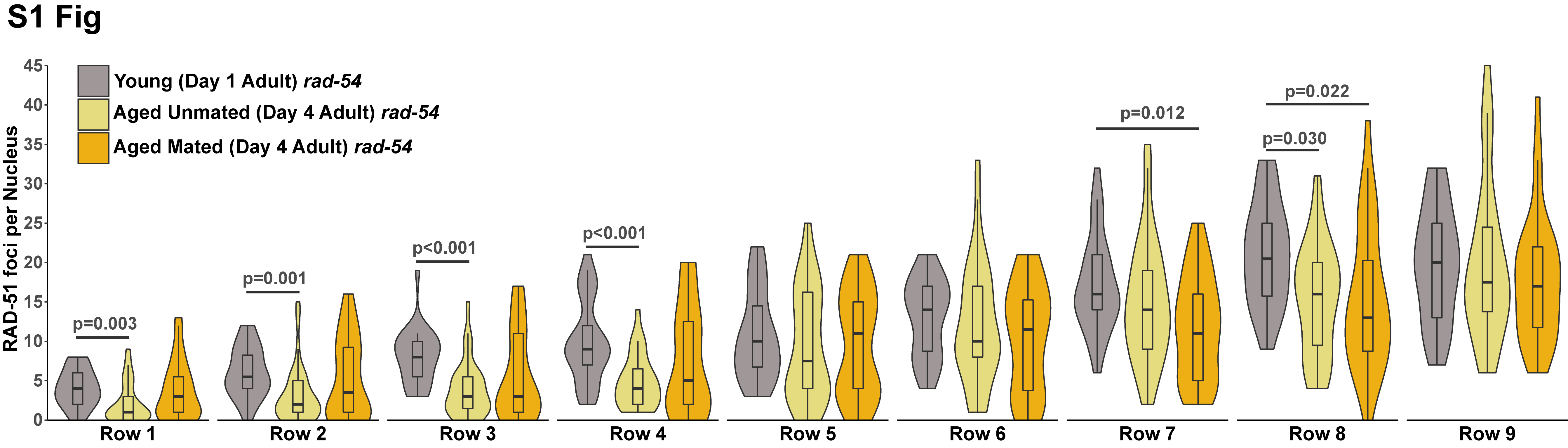

Supplement: S1 Fig — Violin and box plots depicting the number of RAD-51 foci in early pachytene germline nuclei of young (day 1 adult) and aged (day 4 adult) mated or unmated animals. Nuclei were quantified based on their positions in rows progressing through the germline on the distal proximal axis. Row 1 indicates the first row of nuclei in early pachytene. P values were calculated by pairwise Mann-Whitney U tests within each row of nuclei with Bonferroni correction for multiple comparisons. For simplicity, only significant (corrected p value ≤0.05) comparisons are displayed. Nuclei were scored from 6 germlines for each age and mating group (young, aged mated, aged unmated) derived from ≥2 experimental replicates. Each violin and boxplot represents an average of 29.5±3.3 nuclei (minimum 24 nuclei, maximum 37 nuclei, median 29 nuclei). Numerical data associated with this figure is presented in S2 Data. (TIF) [file pgen.1010282.s001.tif]

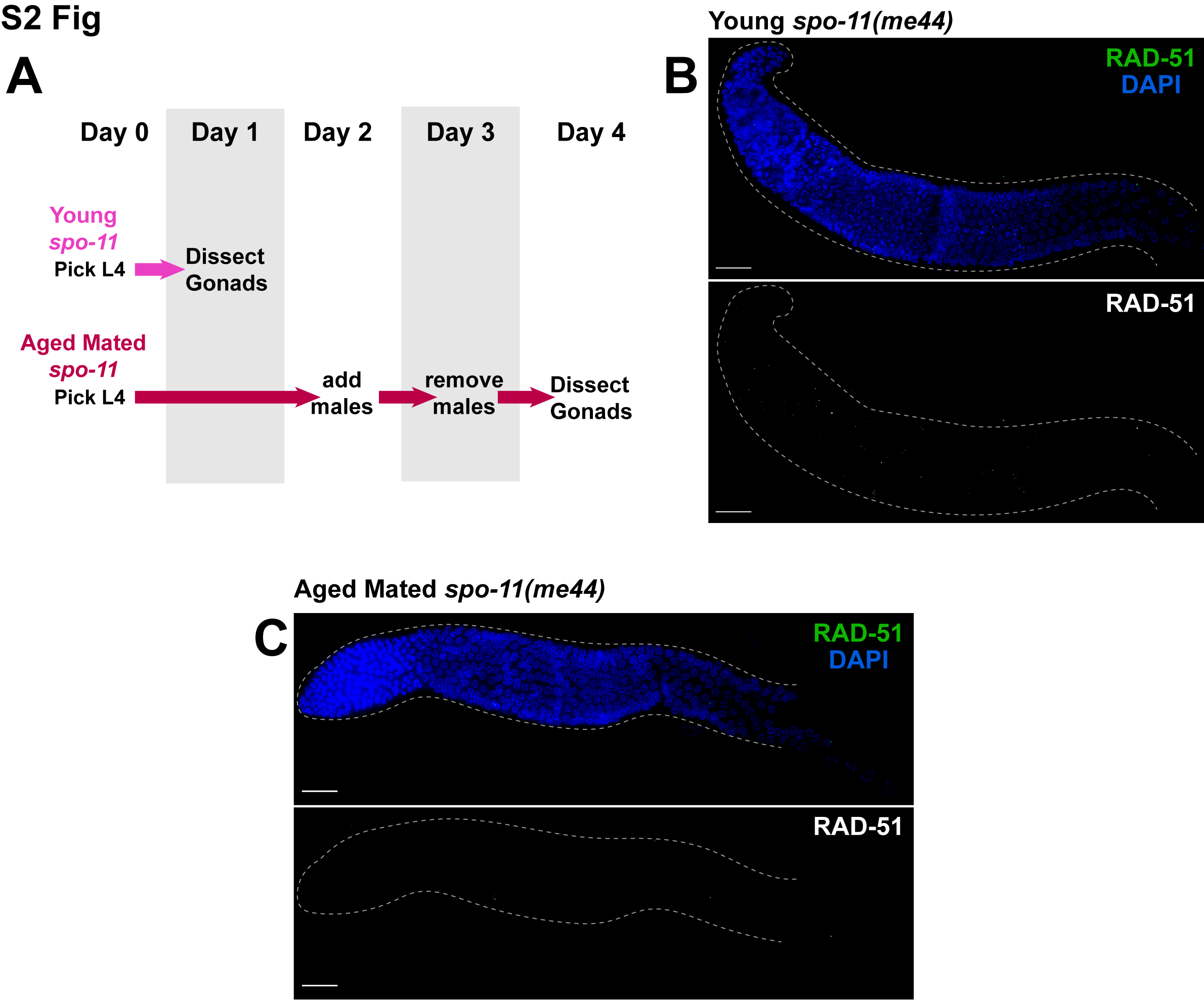

Supplement: S2 Fig — A) Schemes used to isolate young (1 day post-L4) and aged (4 days post L4) worms for experiments. B-D) Representative whole gonad images of RAD-51 stained germlines from young and aged mated spo-11(me44) mutants. Top panels show merged images of both RAD-51 and DAPI, while lower panels show only RAD-51 staining in greyscale. Gonads are oriented with the distal mitotic tip on the left and the end of pachytene on the right. Gonads are outlined with grey dashed lines and scale bars represent 20μm. (TIF) [file pgen.1010282.s002.tif]

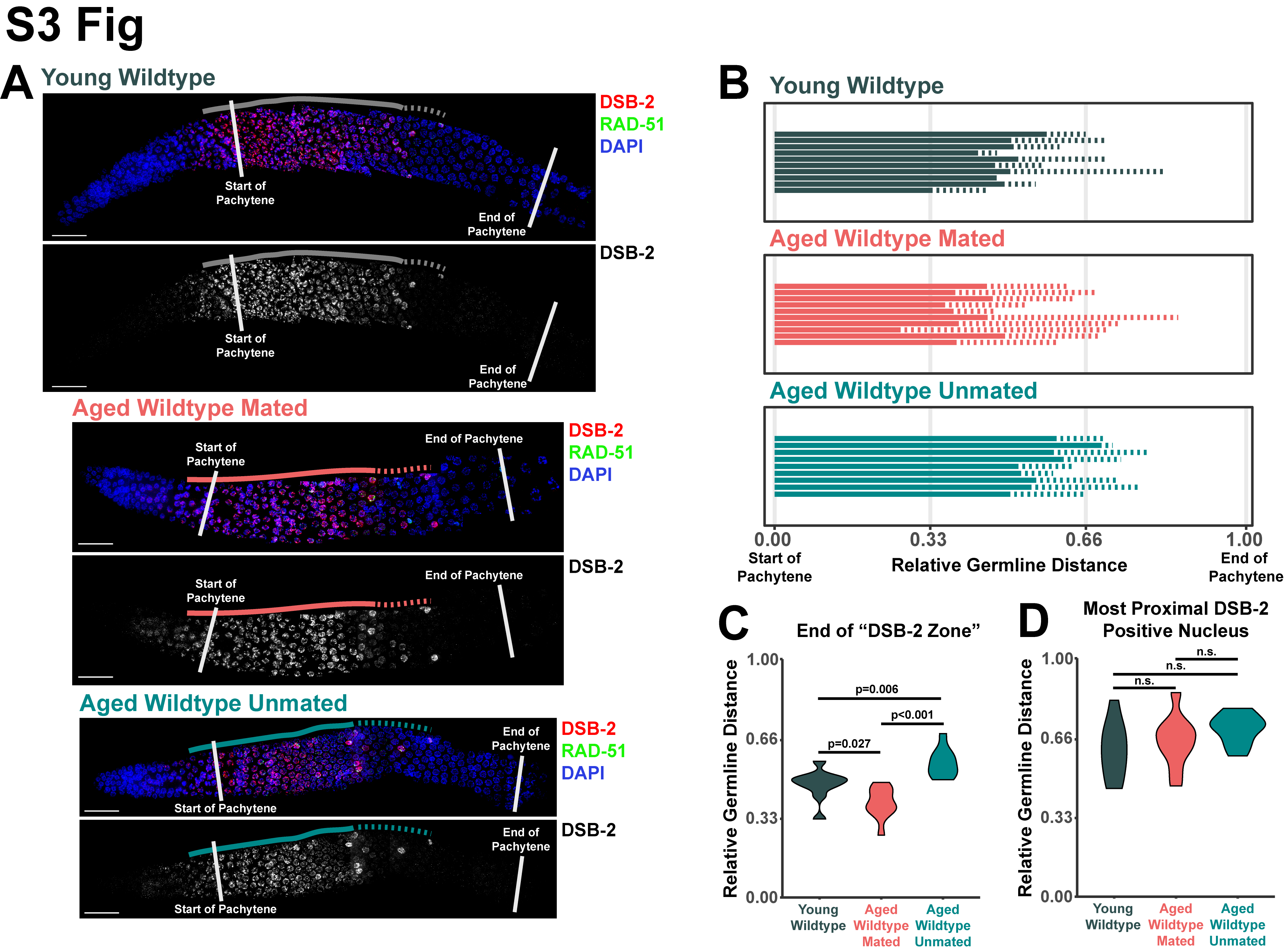

Supplement: S3 Fig — A) Representative images of germlines stained with DSB-2. Solid lines indicate the “DSB-2 zone”, defined as the region of the germline in which >50% of nuclei are stained with DSB-2. Dashed lines extend from the end of the DSB-2 zone to the most proximal nucleus which has DSB-2 staining. Scale bars represent 20μm. B) Line plot representing the quantification of DSB-2 staining in young, aged mated, and aged unmated N2 hermaphrodite germlines. For specific maintenance schemes of these groups, see Fig 1A and Methods. Each horizontal line represents the portion of a single germline which contains DSB-2 positive nuclei. Solid lines represent the “DSB-2 zone”, while dashed lines extend to the most proximal germline position at which 1 or more nuclei is marked with DSB-2. C-D) Violin plots comparing the end of the DSB-2 zone and the final position of DSB-2 positive nuclei in young, aged mated, and aged unmated germlines. P values were calculated by Mann-Whitney U test with Bonferroni correction for multiple comparisons. Numerical data associated with this figure is presented in S7 Data. (TIF) [file pgen.1010282.s003.tif]

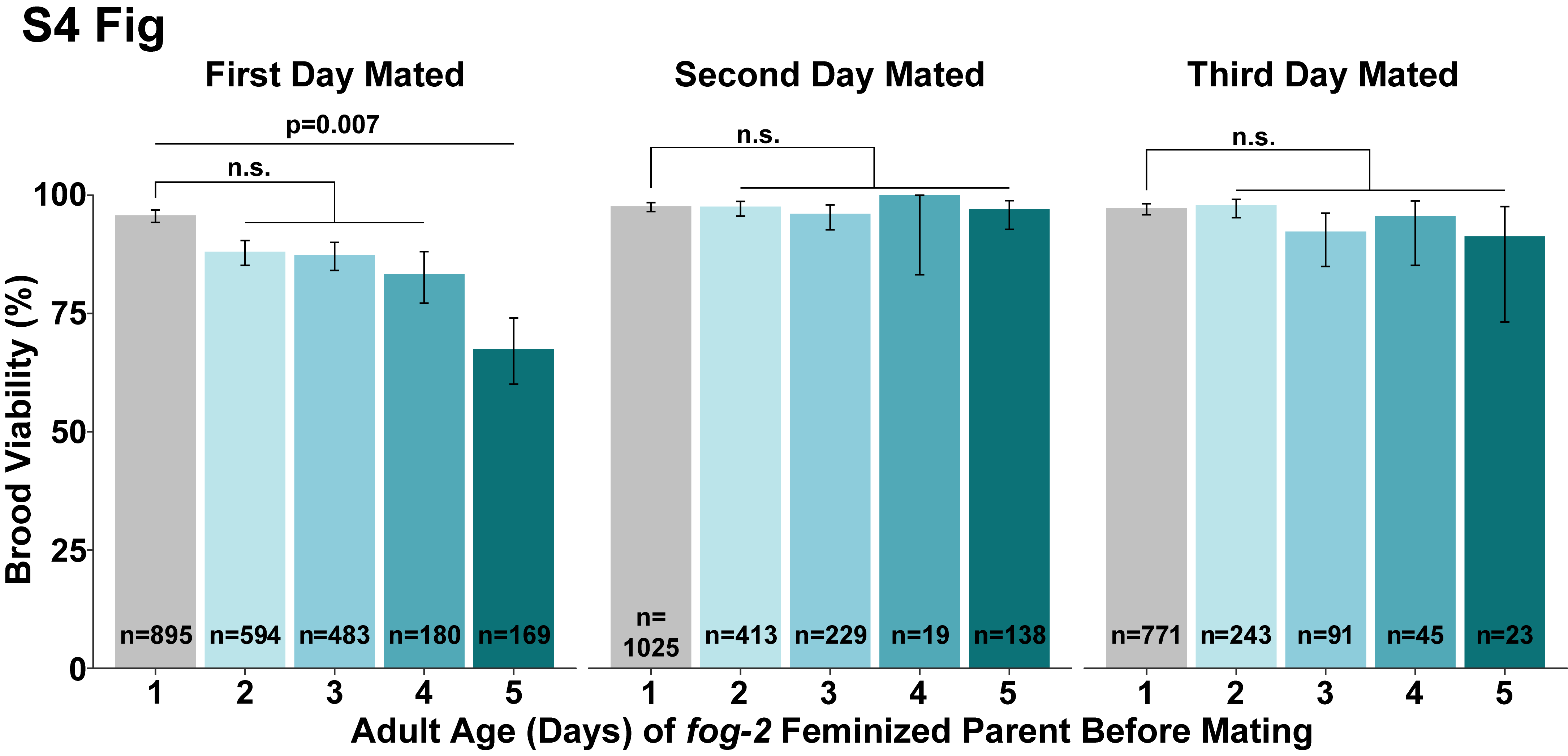

Supplement: S4 Fig — Bar plots representing the population brood viability of mated fog-2 mutant females. In this experiment, fog-2 females were maintained in the absence of males for 1–5 days post-L4 stage (X axis) and were then successively mated for three days. The survival rate of ovulated progeny is depicted for each successive day of mating ‘First/Second/Third Day Mated’ (see Materials and Methods). Error bars represent 95% Binomial confidence intervals. P values were calculated by Fisher’s Exact Test. N values indicate the total number of live progeny and dead eggs scored. P values >0.05 are indicated as n.s. (not significant). Numerical data associated with this figure is presented in S3 Data. (TIF) [file pgen.1010282.s004.tif]

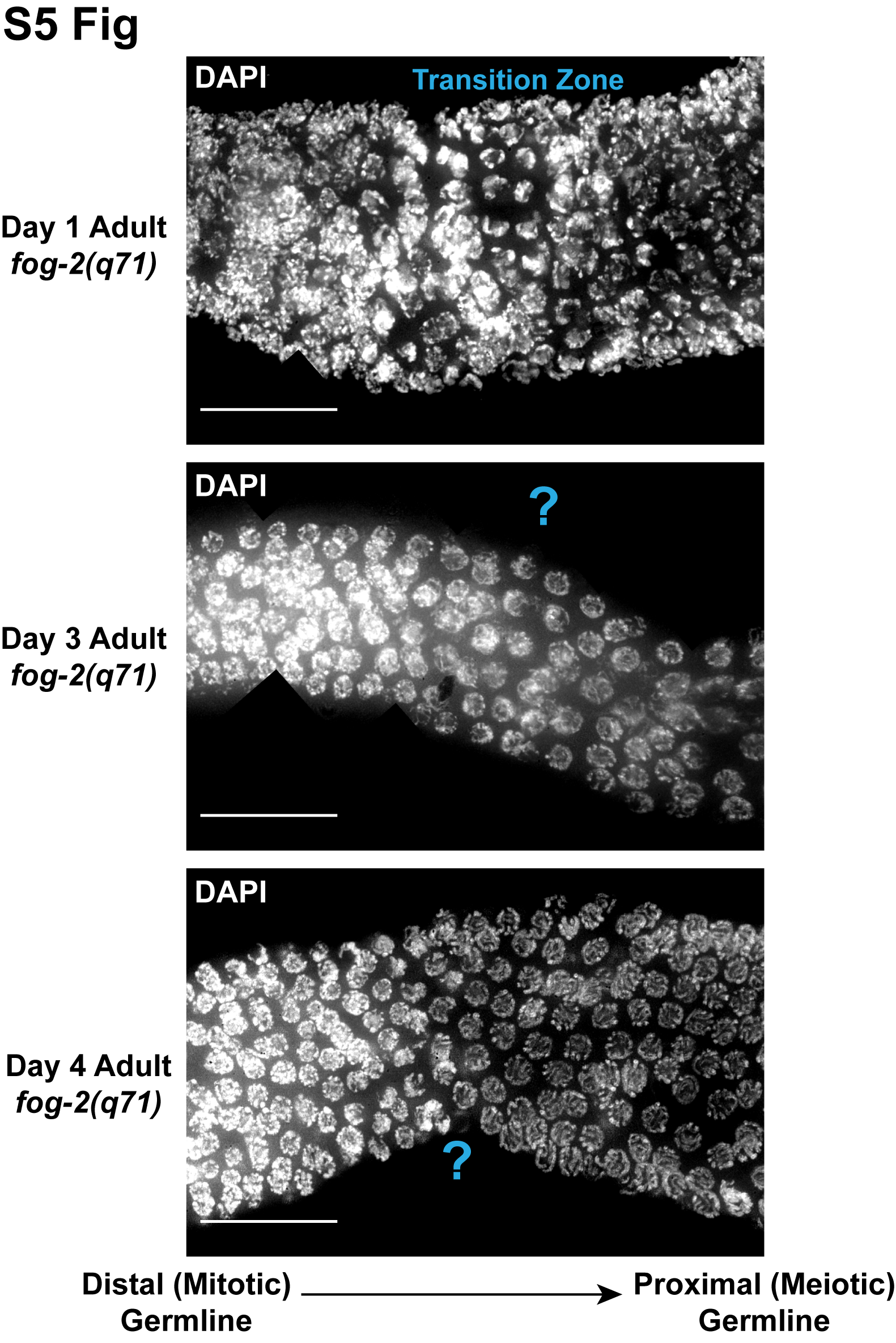

Supplement: S5 Fig — Representative images of fog-2(q71) feminized mutant germlines from animals 1, 3, or 4 days post-L4. The transition zone is marked with a solid blue line in germlines which have crescent shaped nuclei indicative of meiotic entry. Dashed lines indicate the regions of the germline presumably bridging the mitotic and meiotic germline in which crescent shaped ‘transition zone’ nuclei are absent. Gonads are oriented with the distal mitotic region on the left and the proximal meiotic region on the right. Scale bars represent 20μm. (TIF) [file pgen.1010282.s005.tif]

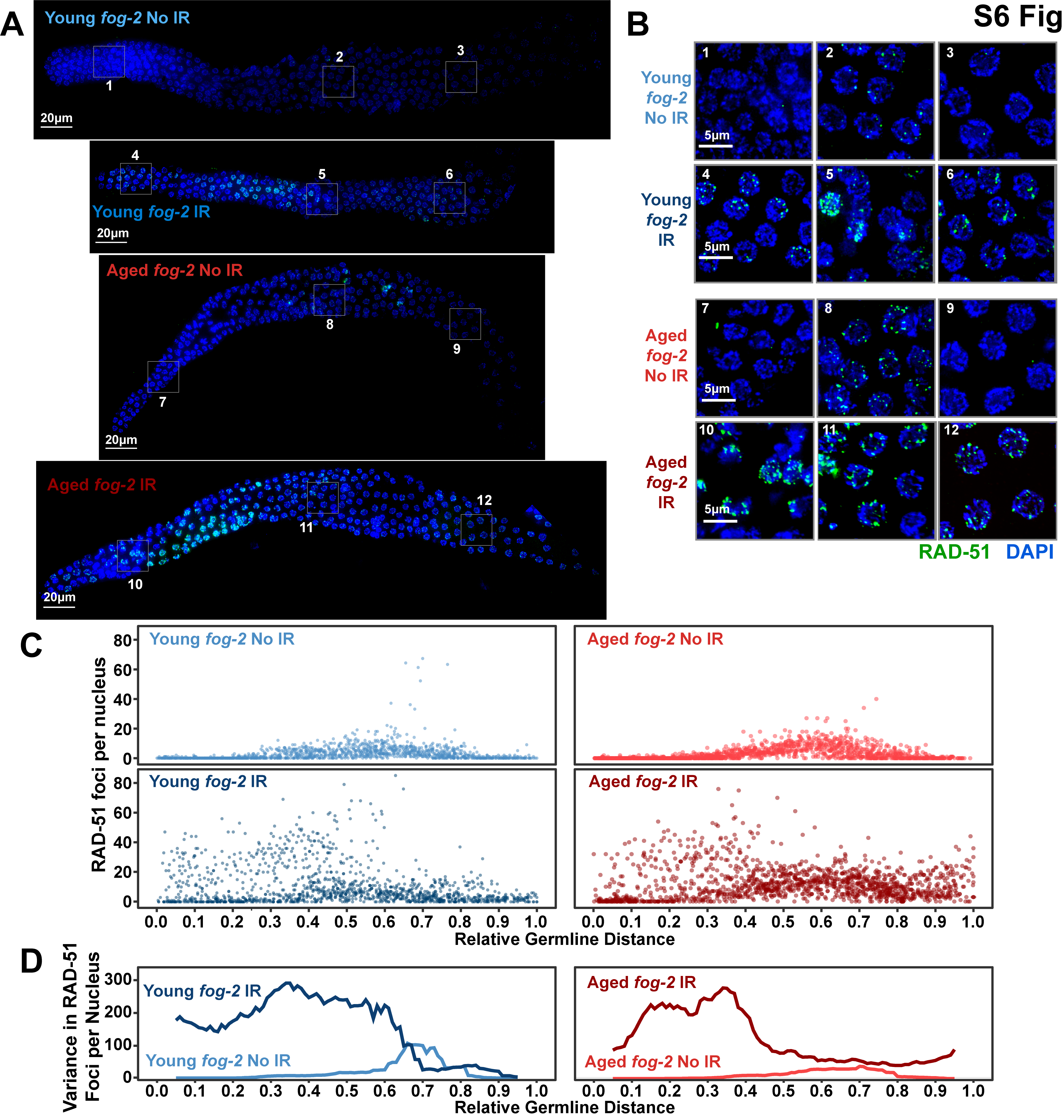

Supplement: S6 Fig — A) Representative images of germlines from young and aged irradiated and unirradiated fog-2(q71) germlines. For specific maintenance schemes of each group, see Fig 3A. Scale bars represent 20μm. Grey numbered boxes indicate inset panels of nuclei displayed in panel B. B) Represented images of subsets of nuclei from germlines displayed in panel A. Numbers on images correspond to the grey boxes in panel A indicating the portion of the germline each image is derived from. Scale bars represent 5μm. C) Dot plots indicating the RAD-51 foci per nucleus in fog-2(q71) IR or No IR young and aged. Each point represents a single nucleus at a given germline position normalized by the premeiotic tip (0) to late pachytene (1) (see Methods). D) Variance in RAD-51 foci per nucleus calculated in a sliding window along the length of the germline where the width of the window is 0.1 germline distance units and the step size is 0.01 germline distance units. Average nuclei quantified in each bin ± standard deviation: Young fog-2 No IR 141.6±26.6, Young fog-2 IR 134.4±27.9, Old fog-2 No IR 148.5±29.6, Old fog-2 IR 142.4±30. Numerical data associated with this figure are presented in S1 Data. (TIF) [file pgen.1010282.s006.tif]

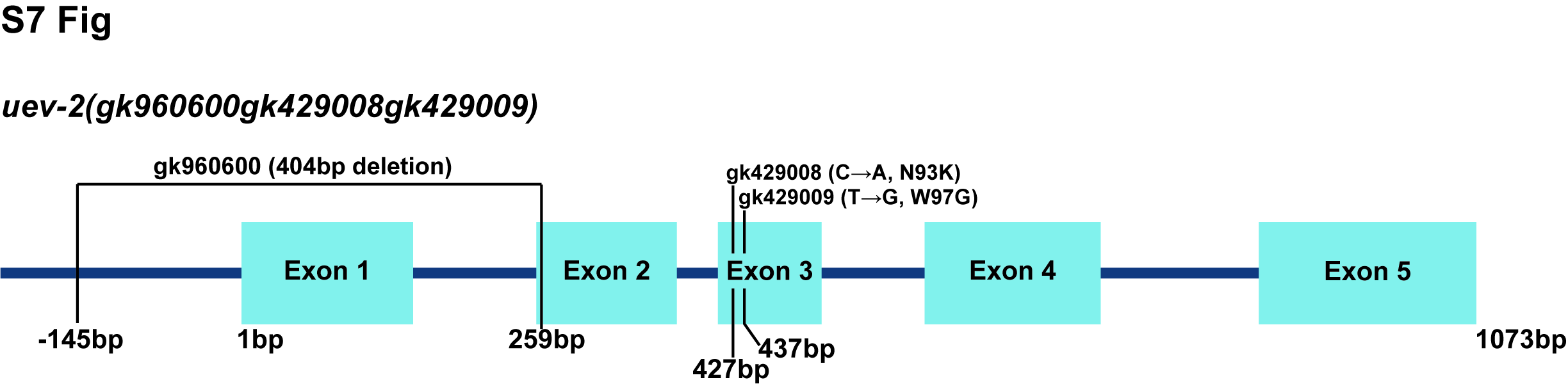

Supplement: S7 Fig — Displayed is a scale cartoon of the uev-2 locus where exons are displayed as boxes and intronic or noncoding upstream sequence is displayed as lines. The gk960600 allele deletes the translation start site and the 5’ intron boundary of exon 2. This lesion generates a frameshift mutation and likely eliminates gene function. Additional point mutations gk429008 and 429009 cause single amino acid substitutions. Base pair distances are indicated relative to the translation initiation site of Exon 1 of the uev-2 coding sequence. (TIF) [file pgen.1010282.s007.tif]
