# Supplementary material for: Aging and sperm signals alter DNA break formation and repair in the C. elegans germline
Source: PLoS Genet. 2022 Nov 7;18(11):e1010282. doi: 10.1371/journal.pgen.1010282 (PMC9671421; doi:10.1371/journal.pgen.1010282)
Supplement: Attachment — Submitted filename: Aging Oocyte Paper Reviews v5.pdf [file pgen.1010282.s015.pdf]

RESPONSE TO REVIEWER COMMENTS:

Reviewer #1:

The manuscript by Toraason et al., characterizes the contribution of oocyte aging and sperm depletion on meiotic DSB formation and processing in the C. elegans germ line. Taking advantage of unmated and mated hermaphrodites and the obligate female fog-2 mutants, the authors carefully tease apart the impact of sperm depletion and aging on RAD-51 kinetics in the germ line. Additionally, the authors provide preliminary evidence that the E2 variant, UVE-2, contributes to the aging phenotype. For the most part, the data is very clear and well presented. The following should be addressed:

1. As the field is limited to RAD-51 as a readout of both DSB formation and processing, it is difficult to tease apart how sperm depletion and aging impact DSB formation vs. processing. I think it would be useful to examine total number of breaks by inactivation of RAD-54 under the different conditions. While I appreciate that this has the added complication of activating signaling pathways to maintain DSB formation, it would still provide important information about DSB formation vs. processing, which is central to many of the conclusions. I also note that while the authors were very careful in the results section to not specifically attribute RAD-51 foci to DSB formation, in the discussion they specifically equate DSB induction with RAD-51 foci.

We thank the reviewer for this suggestion to directly test the extent of DSB induction in differing germline contexts. Although we have some major hesitations regarding the accuracy of DSB quantification using the *rad-54* mutant in either a young or aged context for any study (as outlined below), we assessed the accumulation of RAD-51 foci in aged *rad-54* mutant germlines in both mated and unmated contexts (Supplemental Figure 1, Lines 385-393). While we imaged the entire RAD-51 zone for the *rad-54* germlines, we only included quantifications for nuclei in early pachytene due to multiple reasons: 1) due to the spatial-temporal organization of the *C. elegans* germline, the nuclei in early pachytene in aged germlines have entered meiosis most recently and therefore will best reflect the DSB induction rates of that aged animal; 2) *rad-54* mutants are known to accumulate DSBs throughout pachytene progression at levels far higher than is thought to be occurring in a wild type context due to altered meiotic checkpoint signaling (*e.g.* feedback loops for DSB formation when there are no CO designated sites being processed), and as such the introduction of this *rad-54* mutation fundamentally alters DNA damage signaling and may potentially impact the aging trajectory of meiotic processes; 3) in aged *rad-54* mutant, we observed variance in both the number of nuclei present and the quality of germline structure/organization, potentially altering the timing of meiotic progression, and therefore also the amount of DSBs accumulated by the end of pachytene in aged contexts and biasing results which might be obtained from this stage; 4) late pachytene RAD-51 foci in a *rad-54* mutant tend to look elongated, which could represent either longer RAD-51 binding substrates surrounding a DSB site or convergence of two RAD-51 foci, which complicates the ability to make clear and accurate calculations of DSB numbers in this stage; and, 5) we do not want other groups to take any quantified late pachytene data out of context from our study to reference in their future studies because of the issues outlined above, we do not want to be associated with any conclusions that are attempted to be made from RAD-51 numbers from the late pachytene region of the *rad-54* mutant. As stated in the supplemental discussion of Rosu, Libuda, and Villeneuve *Science* 2011 where RAD-51 foci counts were performed in young adult *rad-54* mutants, "differences in sensitivity of detection,

coupled with the possibility that DSBs might be altered in a mutant with impaired progression of recombination, make [RAD-51 staining in a *rad-54* mutant] difficult to infer DSB numbers with precision." In concordance with our wildtype data, our analysis of early pachytene RAD-51 foci in *rad-54* mutants for these revision experiments indicate that DSB induction was delayed specifically in aged unmated germlines, which had depleted their self supply of sperm, and non-aged mated germlines. This result parallels prior analysis of RAD-51 foci accumulation in aged *rad-54* mutants performed by Raices *et al. Front. Cell Dev. Biol.* 2021.

2. UVE-2: The authors show that mutation of uve-2 leads to a "premature aging" phenotype with respect to RAD-51 foci. It would really strengthen the paper if the authors examined localization/expression of UVE-2 in the germ line over time – does UVE-2 expression decrease with age? I also think it will be very important to measure the extent of over-expression of UVE-2 from the pie-1 driven construct in comparison to young and old wild-type expression given that the effect observed was subtle.

We appreciate this suggestion by the reviewer. Previous analysis of gene expression over *C. elegans* lifespan by Mansfeld *et al. Nat. Comm.* 2015 has found that *uev-2* expression increases with age. In the revised manuscript, we now specifically mention and cite these published results on *uev-2* expression and provide a possible interpretation as to how our results relate to this published data (Lines 743-747)

3. In the discussion the authors suggest that the contradictory finding concerning SPO-11-dependence between this study and a previous study could be due to the nature of the SPO-11 allele and/or strain background differences. I recommend including the molecular details of the two alleles directly in the discussion.

We thank the reviewer for this suggestion. Additional context has been added to the discussion (Lines 708-720).

4. I really like the figures with respect to RAD-51 and the thorough statistics presented. Just a couple of comments: In figure 1, wouldn't n.d. be better as n.a. (not applicable)? In figure 5, the statistical comparison between young uev-2 and wt, should be blue as the p value is 0.043.

We appreciate the suggestion from the reviewer to use "n.a." rather than "n.d." for inappropriate statistical comparisons in our p value heat maps in Figure 1 and have updated the figure accordingly. We additionally thank the reviewer for catching the color error in Figure 5 and have updated this figure as well.

5. I had a hard time deciphering supplemental figure 3: if fog-2 was mated on the second day, why is there values for day 1 brood viability (etc)? I am obviously missing something and suggest that the authors clarify the experimental set up.

We appreciate the reviewer identifying confusing elements in our text regarding this fertility data. The age indicated on the X axis of Supplemental Figure 4 indicates the number of days the parent *fog-2* female was maintained in the absence of males before

she was mated. Each female was then successively mated over the course of three days, represented by the ‘First/Second/Third Day Mated’ groupings of bars. To make this figure clearer, we have amended the X axis legend of Supplemental Figure 4 and have added descriptive text to its corresponding figure legend.

Reviewer #2:

Summary
In this manuscript, the competence of meiotic recombination (DNA break initiation and repair) in C. elegans oocytes was assessed as a function of organismal age and history of exposure to sperm, and found to correlate significantly with both factors in distinct ways. Depletion of sperm in organisms that once held sperm was shown to lead to reduction of break initiation, while age alone irrespective of sperm history led to delays in break repair. Since age and sperm depletion are normally precisely correlated in C. elegans hermaphrodites, the attempt to decouple these two causes was a great idea and has succeeded in bringing new understanding to the causes of decreasing oocyte quality. The manuscript leads naturally into future work where the mechanisms of how age, sperm depletion, and other factors could affect DNA repair may be elucidated.

Critiques

1 - The data are of high quality, in general well-controlled and in sufficient biological replicates, and the statistics appear properly done and corrected for multiple comparisons where appropriate. The method of straightening gonads and measuring foci in sliding windows of normalized distance, rather than the standard practice of going straight to bins, allows a more comprehensive look at the course of foci appearance and disappearance, and should be more widely adopted. One concern I have with the method as described is the reliance on automation and in particular the elimination of nuclei that overlap others (Methods 260-261). It would help the appraisal of the data to be told how many nuclei are typically thus eliminated, and whether there is any possibility that elimination is non-random with respect to RAD-51 focus number. Even if this information is available in the first author's earlier method publication, it would be good to make it explicit here as well. It would defeat the purpose of automation to do this for every single gonad, but a statement clarifying this elimination step and the possibility for bias would be useful.

We are delighted to hear the reviewer’s thoughts on our data visualization, as well as their feedback on potential bias in our automation approach in quantifying RAD-51 foci. In our previous publication on this method (Toraason *et al. Genetics* 2021), we examined RAD-51 foci counts in diverse genetic contexts and demonstrate that our approach enables accurate and even sampling of nuclei across the germline. To clarify this previous work, we have amended the text in the Materials and Methods to delineate these key points (Lines 273-276).

2 - A question/concern about the nature of the meiotic spatiotemporal gradient in *fog-2* animals: The IR experiment showing defects in DNA repair in aged *fog-2* feminized germlines examines DNA breaks that have not been repaired even after 2 days. Normally the time from S phase to pachytene is 24-48 hours (Jaramillo-Lambert et al.) so the expectation for wild-type germlines would be that the pre-pachytene stages should not show IR-induced damage after 48 hours. The introduction (385-) explains that "Due to the absence of signaling from sperm in *fog-2* mutants, both germline stem cell proliferation and meiotic progression are halted, such that meiotic oocytes are held within the gonad" which explains why the damage is still visible. But in

Results (457–) it is stated "aged irradiated germlines maintained a median elevation of ~6-10 RAD-51 foci per nucleus (germline position 0.5-1.0, Figure 3C). This result indicates that aged fog-2 germlines exhibit DNA repair defects specifically in nuclei at later stages of meiotic prophase I.", here explicitly identifying late germline position with late stages of meiotic prophase I. My question (after all this setup) is, to what extent do we know that proximal position in the germline reliably indicates "late meiotic prophase", when all cells in the gonad are old enough to be at least in mid-pachytene? As you show that the transition zone is missing (Sup. Fig.4), it seems that meiosis does progress in these halted cells. So, can the higher RAD-51 foci over background in irradiated *fog-2* aged germlines be properly attributed to the temporal stage of prophase, or could it perhaps be an effect only of spatial position? This experiment just made me think about the assumptions involved in doing the usual spatiotemporal analysis an essentially static gonad, so I think it would be good to guide the reader through the logic in a little more detail.

We appreciate the reviewer's thoughtful interpretation of our results. While the spatial organization of prophase I in the germline becomes disrupted with age in *fog-2* mutants, as evidenced both by disappearance of the transition zone and expansion of the RAD-51 window in old *fog-2* females, we also observed that the extent of nuclei which had bright COSA-1 foci was not extended in old germlines (Figure 4). This result suggests that not all meiotic events occur independent of germline position and that some steps in meiotic progression may be spatially limited within the gonad. As such, we do not believe that progressively distal nuclei within the *fog-2* germline are completing crossover designation and moving into late pachytene with time. With our current data, however, we agree that we are unable to decouple the specific contributions of meiotic stage and germline position, so we have amended the text to reflect this point (Lines 523-525).

3 - The conclusion that sperm *depletion*, not sperm absence or age, is causing a reduction in break initiation in early pachytene receives support from (1) the difference between (aged and sperm-depleted wild-type) versus (aged and mated wild-type) animals, and (2) the difference between (aged and unmated wild-type) and (aged and unmated *fog-2*) animals (e.g. discussion 567-569), the latter having never encountered sperm. The evidence would be stronger still if *mated but sperm-depleted aged fog-2* animals could be examined, since the prediction is that they would show the same DSB initiation defects as wild-type sperm-depleted animals. Technical considerations may render this experiment unfeasible, and this result is not necessary to accept the conclusion, but for the manuscript's *fog-2* results to count as evidence I think it must be assumed that *fog-2* germlines would have shown reduced RAD-51 foci had they been transiently exposed to sperm.

This suggestion from the reviewer is an excellent test of the model we propose in this manuscript. We have assessed RAD-51 foci in *fog-2* worms which were mated and then allowed to deplete this supply of sperm (Figure 2, Lines 486-496; Materials and Methods, Lines 201-207). We observed that the levels of RAD-51 foci as well as the RAD-51 zone was notably reduced in these old sperm depleted germlines, paralleling our results in self-sperm depleted wild type contexts. This result strongly supports our proposed model and indicates that sperm depletion is sufficient to downregulate RAD-51 levels in the *C. elegans* germline.

Minor comments:

Lines 338-340 "During early pachytene, the amount of RAD-51 foci per nucleus was similar between aged mated germlines and young germlines…Young germlines, however, accumulated a higher total number of RAD-51 foci per nucleus…" the second part here should contain a reference to the later stage it refers to, otherwise it may confuse the reader (is it similar or higher?)

We appreciate the reviewer pointing out places where we could be clearer in referencing our figures. In answering another comment, we have amended the draft to include specific values for RAD-51 foci per nucleus in the text whenever we reference a specific section of a plot, and in doing so we have incorporated explicit indication of which Bins we are referring to within the text.

Fig. 1, 5, and 6 have mislabeled panels (A,B,B instead of A,B,C).

We are grateful the reviewer found this error. The panel labels have been amended for all figures.

Fig. 2, the "RAD-51 zone" is defined once in the text and twice in the legend with slightly different wording each time; although the different descriptions are mostly consistent it would be better to define it only once.

We thank the reviewer identifying these redundancies, and we have simplified the manuscript by only describing the RAD-51 zone in the text.

Fig. 3, panel B shows mean focus number per nucleus, consistent with Figs. 1,2,5, and 6, but panel C (the subtraction between +/-IR) uses the median; if this is necessary, it should be given an explanation.

The reviewer raises a valid concern about which summary statistic is appropriate for this analysis. As the irradiated germlines exhibited very high variation and some extreme outliers in RAD-51 foci counts (Supplemental Figure 6), we were concerned that the mean levels of DSBs may be inflated by these outliers. We therefore instead employed the median RAD-51 foci as our metric for this analysis specifically to better approximate the DSB levels within nuclei as a population. We have clarified this point in the text (Lines 516-518).

Fig. 3, the nomenclature for young vs. aged is changed somewhat from Fig. 2 ("young" Fig. 2 animals are dissected at 1 day post-L4, while "young" Fig. 3 animals are dissected at 3 days) and could lead to confusion; a change in naming might help, but if no alternative seems better, then just explaining this in the Figure legend would suffice.

We appreciate the reviewer identifying this potential point of confusion, and we have amended the figure legend (Lines 816-818).

Fig. 6: it should be stated in the legends that the data for Young Wildtype and Aged Wildtype Mated are the same as Fig. 5.

We thank the reviewer for this suggestion, and we have amended our figure legends to specifically point out where data has been used multiple times.

Reviewer #3:

Toraason et al., investigated the influence of two factors, sperm and reproductive aging, on oocyte quality in C. elegans. They specifically analyzed double-strand breaks (DSBs) induction and repair during meiotic prophase I in oocytes and how they are affected by aging, sperm and their combination. They dissected these factors by taking advantage of C. elegans hermaphrodites (WT) that produce oocytes and sperm, and obligate female mutants (fog-2) that produce only oocytes. Experimental design also accommodated the difference between complete lack of sperm exposure vs sperm depletion. The WT and obligate female mutants were used to investigate the role of sperm and aging. Authors concluded that sperm depletion instead of the complete absence of sperm downregulates DSBs suggesting that loss of signals from sperm causes deficiencies in DSBs induction and repair. Authors also confirm that reproductive aging is the main factor responsible for the DNA repair deficiency in the aged germline. In the second part of the manuscript authors show that the E2 ligase variant UEV-2 which has previously been implicated in DSB repair efficiency is important for DSBs in young germlines and that aging may affect its efficacy. Overall, the experiments that are reported are well executed and represented. However, some analyses seem incomplete as listed in major points below (for example radiation experiment is only done in fog-2 germlines which means only in the absence of sperm). Missing experiments are needed to strengthen the manuscript unless authors provide explanation to why they were not included or would not be informative. The notion that sperm signals are regulating oogenesis is known but the authors designed an original approach to dissect it genetically. Results will have a high to moderate importance on reproductive research in C. elegans. However, the relevance for human reproductive aging research is not clear. Two aspects of this model system are not relevant for human: 1) sperm has no effect on mammalian meiotic recombination; 2) Meiotic recombination in oocytes takes place in utero and aging affects meiotically arrested oocytes and their ability to repair DSBs. Authors did not investigate oocytes arrested in diakinesis or mature oocytes which are equivalent to human oocytes<u>. To increase the importance and relevance, authors could provide stronger explanation how their findings inform aging research in other system.</u> Minor and major points for authors to consider are listed below.

Results:
Minor:

1 - General comment: it would be helpful if authors provided results as numbers in the text (i.e. number of foci quantified) rather than always referring reader to a figure. For example:

- Line 342-346: “ were greatly decreased throughout early pachytene as compared to both young and aged mated germlines (Figure 1B-C, Bins 2-3 Mann-Whitney U test $p<0.001$).
- Line 469-470 : “with a subtle but significant increase” please provide the values in text.

We appreciate this suggestion from the reviewer, and have included specific values for the mean and standard deviation of RAD-51 foci per nucleus in the text.

2- <u>Major</u>:
• Lines 377-379: Authors conclude that “the extent of DSB-2 marked pachytene nuclei is

influenced both by aging and by the absence of sperm" giving both factors equal weight while in fact the effect of age was "subtle". I would recommend rephrasing this conclusion to represent the data.

We thank the reviewer for pointing out this discrepancy in our language, and we have amended the text (Lines 427-429).

• Line 380-433: Why were fog-2 germlines not compared to N2 wildtype germlines? To determine the impact of sperm production n young and old germlines I would expect comparison between young WT vs fog-2, old WT mated vs fog-2 old mated and old WT unmated vs fog-2 old unmated. Provide explanation why these comparisons were not made.

We agree with the reviewer that comparing RAD-51 foci between wild type and *fog-2* germlines is a potentially very interesting analysis. However, the differing physiology of arrested and proliferating germlines creates a challenge in delineating which nuclei are accurately comparable/equivalent between wild type and *fog-2* germline contexts. In particular, the absence of the transition zone as well as the known delay in nuclei progression in aged *fog-2* mutants precludes germline position alone as a metric by which to assess which nuclei in wild type and *fog-2* germlines are comparable to assess their levels of DSBs. To avoid making erroneous conclusions from potentially inappropriate comparisons, we have refrained from directly comparing aged wild type and *fog-2* germlines. Nonetheless, our additional experiments in mated *fog-2* worms (Figure 2, Lines 486-496) have provided additional insights into the effects of mating in the *fog-2* context, addressing similar questions to those posed by the reviewer.

• Lines 432-433: the model should be validated by analyzing DSB induction in fog-2 mutants mated to males

We think that this experiment is a great idea, and we thank the reviewer for this suggestion. We have assessed RAD-51 foci in *fog-2* worms which were mated and allowed to deplete this supply of sperm (Figure 2, Lines 486-496). We observed that the levels of RAD-51 foci as well as the RAD-51 zone was notably reduced in these old sperm depleted germlines, paralleling our results in self-sperm depleted wild type contexts. This result strongly supports our proposed model and indicates that sperm depletion is sufficient to downregulate RAD-51 levels in the *C. elegans* germline.

• Lines 447-449: Is this sensitive subpopulation specific to fog-2 mutant or also exist in WT? Again, explain lack of comparison with WT irradiated.

The reviewer raises an interesting question, however, assessing the long-term impacts of irradiation on wild type germlines is difficult due to the continuous proliferation and progression of meiotic nuclei through the tissue (as noted by Reviewer 2). We believe this sensitized subpopulation is interesting and may warrant investigation in a future study dedicated to the specific nature of these nuclei.

• Lines 460-461: Without comparison with WT irradiated germlines I', not sure authors can conclude that" that DNA repair efficiency is altered in aging germlines independent of any

signals from sperm” because analysis was performed only in females without sperm. If the conclusion is valid, then similar results should be seen in aged hermaphrodites mated and unmated after IR.

We thank the reviewer for raising this important comparison to wild type. We did not focus on DSB repair following ionizing radiation in aged germlines which are undergoing proliferation and germline progression because these experiments were previously performed in work by another group (Raices *et al. Front. Cell Dev. Biol.* 2021). Raices *et al.* demonstrated that recombinase loading and DSB repair was delayed following irradiation in *spo-11* mutants during aging. Our results are complementary to and produced results which parallel these previous experiments. To better highlight this previous work, we have amended the text in our results to cite the Raices *et al.* findings (Lines 525-527).

• Lines 479-480: Similarly, can the CO formation be analyzed in fog-2 mutants depleted of sperm to validate the conclusion?

We agree with the reviewer that this is an important experiment, and we have assessed GFP::COSA-1 foci in aged *fog-2* females which have been mated and allowed to deplete their supply of sperm. We found that old sperm depleted *fog-2* germlines also exhibited altered numbers of GFP::COSA-1 foci. These results are displayed in Figure 4 and are discussed in Lines 540-543).

• The analysis of uev-2 mutants does not include analysis of crossovers using COSA marker. It would improve the manuscript if impact of aged-like-DSB repair was tested for consequences in Crossovers and oocyte quality.

We thank the reviewer for raising the question of a potential function for UEV-2 in regulating the formation of interhomolog crossovers. To determine if *uev-2* is required for normal meiotic recombination, have assessed the fertility and rate of male progeny (indicative of chromosome nondisjunction) in *uev-2* mutants. We found that *uev-2* mutants do not have reproductive defects relative to wild type (Lines 571-577), indicating that *uev-2* is not required for crossover formation.

Discussion:
Minor:
• Line560-561: Refer to actual data figure or specify that this is referring to a model.

We appreciate the reviewer for this suggestion, and have amended the text accordingly (Lines 661-662)

Major:
• Line569-571: Without data from fog-2 mated females I don’t think authors can conclude that mating has no effect. Is there a male mutant that doesn’t have sperm but still mates with females? This could test how mating may affect DSB induction .

We appreciate the reviewer's feedback on this discussion point. We agree that sperm-independent effects associated with mating likely have impact on hermaphrodite reproduction and potentially DSB formation, but merely meant to suggest that our results imply that sperm depletion is a primary factor in regulating DSB induction. We have altered the language of this discussion point to emphasize this conclusion (Lines 667-673).

• Line 572-573: This is also different than what happens in human oocytes. Comment of this in the context of human relevance.

We thank the reviewer for this suggestion and agree that additional discussion is warranted for *spo-11* independent DSB induction in aged oocytes. As the reviewer indicates, our findings in *spo-11* mutants likely are primarily relevant to the *C. elegans* system. We have therefore expanded upon our discussion of this and focus on the potential basis for the discrepancy in the results which have been found in *spo-11(me44)* and *spo-11(ok79)* mutants (Lines 708-720).

• Line 578-591: Authors provide speculative hypothesis why these changes in DSB induction may be occurring but don't discuss how besides implicating insulin/insulin-like growth factor. Why specifically DSB? Why only reduction/delay and not complete cessation?

We think that these questions are all very interesting, and appreciate the reviewer for raising them. We have expanded our discussion on the potential mechanisms through which DSB induction might be differentially regulated during sperm depletion (Lines 690-707).

• Comment on early induction of DSBs in Aged mated compared to young (figure 1, Bin 1). In other words, comment on the significance in aged mated to both aged unmated and young. Is it due to the presence of exogenous sperm? As male sperm is known to outcompete hermaphrodite sperm so maybe male sperm may also have a "strong signal" for DSB induction?

We appreciate the reviewer's observation of this phenotype, and have amended the text to include some discussion of it (Lines 676-679).

Abstract:
Minor:
• Line 53 input short name "(C. elegans)-

The accepted convention for binomial nomenclature (also known as binary names) is that the abbreviated binomial name of a species with the generic name abbreviated should not be included in parentheses immediately after the full generic name and specific epithet (e.g. full genus and species name) when that organism is initially introduced in a manuscript. Instead, the convention is that the abbreviated epithet is utilized in the next text reference to the organism. The American Society of Microbiology describes this accepted convention as well as other various conventions for field

standards on their society journal page. In consideration of this accepted convention, we have decided to maintain the binomial nomenclature as is in the text.

• Line 59: … meiotic prophase I progression

We have implemented this edit (Line 59)

Author Summary
Minor:
Please see our response to the comment regarding Line 53.

Methods:
Minor:
• Lines 163 and 161: N2 (wild type) repeated

We thank the reviewer for catching this error, and have removed the duplicate strain.

• Lines 166-172: Make another section may be labeled "experimental assay" and that can also include the fog-2 Brood viability assay

We appreciate the reviewer's suggestion for clarity in reporting our experimental methodology. To emphasize the conditions under which aging occurred in our experiments, we have added a specific Methods subheading for 'Aging conditions' (Lines 192-207). Traditionally, brood viability assays are reported separately from culture conditions in the *C. elegans* literature, so we have refrained from combining this information with the aging culture conditions to match the conventions of the field.

• Lines 222, 290: are "worms" needed after C. elegans?

We appreciate the reviewer's comment on this wording, and have amended the text at these sites to instead specify '*C. elegans* strains'.

• Lines 250-253 Provide suppliers and catalogue numbers for commercial antibodies.

We have added the requisite information for the commercial antibody in this study (Lines 260-265)

• Mann-Whitney U test is not mentioned in statistical methods description.

We appreciate the author's thoughts on clarity in our statistical methods. We include a statement in the statistics section of the methods pointing the reader to the text and figure legends for specific statistical tests used, and we utilize thorough labeling throughout the manuscript for clarity in the statistics being applied.

Figures:
Figure 1:
• Define n.d

We thank the reviewer for pointing out this undesignated abbreviation, and have defined it in all figure legends where appropriate.

• Line plot mislabeled as panel B

We are grateful for the reviewer for finding this error. The panel labels have been amended for all figures.

Figure 5 and 6:
• Line plot mislabeled as panel B

We truly appreciate the reviewer identifying this error. The panel labels have been amended for all figures.

Have all data underlying the figures and results presented in the manuscript been provided?
Large-scale datasets should be made available via a public repository as described in the *PLOS Genetics* data availability policy, and numerical data that underlies graphs or summary statistics should be provided in spreadsheet form as supporting information.

Reviewer #1: Yes

Reviewer #2: **No:** I could not find spreadsheets in the main or supplemental figures containing numerical data that the RAD-51 focus graphs were based on.

We now include excel spreadsheets delineating the raw numerical data used in this study. These files include our COSA-1 counts in *fog-2* mutants, the brood viability counts in *fog-2* mutants, the brood viability and incidence of males counts in wild type and *uev-2* mutants, DAPI body counts in wild type and *uev-2* mutants, RAD-51 counts in *rad-54* mutants, and RAD-51 counts in wild type, *uev-2*, *fog-2*, and *pie-1p::uev-2* mutants.

Reviewer #3: Yes
