# Supplementary material for: Aging and sperm signals alter DNA break formation and repair in the C. elegans germline
Source: PLoS Genet. 2022 Nov 7;18(11):e1010282. doi: 10.1371/journal.pgen.1010282 (PMC9671421; doi:10.1371/journal.pgen.1010282)
Supplement: Attachment — Submitted filename: ToraasonRevision2ResponseReviewers.pdf [file pgen.1010282.s016.pdf]

<u>Response to Reviewers for Toraason, Adler, and Libuda (Revision #2)</u>

1) The authors refer to UEV-2 as a variant E2 ubiquitin ligase – based on all the information I can find (which wasn't very much) UEV-2 is an E2 conjugating enzyme.

We appreciate the reviewer's diligence pertaining to our specific terminology and have updated the text to reflect this more accurate description of UEV-2 as an E2 enzyme variant.

2) A point that wasn't addressed was the extent of overexpression of UEV-2 driven by the pie-1 promoter. I still think this is important– is it 2-fold, 10-fold, 100-fold overexpressed? This is relevant to interpreting the observed phenotype.

We agree with the reviewer that the specific levels of *uev-2* may underpin the extent of the phenotypes we observed in our *pie-1p::uev-2* overexpression line. To <u>contextualize our observed phenotypes within the known levels of *uev-2* expression and the expected strength of our overexpression construct, we have expanded upon our discussion of the *pie-p::uev-2* overexpression results (Lines 734-740).</u> While *pie-1* has been commonly used as a promoter to drive germline transcription in *C. elegans*, characterization of transgene expression levels using this promoter either in single copy or in multiple copy array or bombardment lines is not widely available in the literature nor standard in the field. Germline-specific RNAseq data indicates that *pie-1* is endogenously expressed at levels >200 fold higher than *uev-2* (Han *et al. BMC Genomics* 2019). Given that we observe some phenotypes (albeit moderate) with *pie-1p::uev-2* overexpression, it very possible that either stronger or weaker promoters may even more dramatically alter the observed phenotypes with our *pie-1p::uev-2* overexpression line (which we mention in the amended text). Since the sensitivity of cells to transcript dosage varies widely between genes (Moriya *Mol. Biol. Cell* 2015), knowing the absolute fold change in *uev-2* expression may not significantly add to our understanding of the biology of UEV-2. With the occurrence of a detectable phenotype from an overexpression construct and the issue that quantitative expression changes may not necessarily inform about the biology, it is not standard in the aging field to quantitate the extent of overexpression for these types of experiments (see Booth *et al., Nature Aging* 2022; Kaletski *et al., Nature* 2016; Templeton *et al., Dev Cell* 2022). With all of these factors in mind, we now mention in the revised text how dosage of *uev-2* during the aging process may be important for DSB repair in aging oocytes. Overall, our data demonstrate that UEV-2 regulates germline DSB repair during aging and provides the rationale for future study to better elucidate its specific functions and inform the phenotypes we observe in this manuscript.
